# Helminth-induced Ly6C^hi^ monocyte-derived alternatively activated macrophages suppress experimental autoimmune encephalomyelitis

**DOI:** 10.1038/srep40814

**Published:** 2017-01-17

**Authors:** Cesar Terrazas, Juan de Dios Ruiz-Rosado, Stephanie A. Amici, Kyle A. Jablonski, Diana Martinez-Saucedo, Lindsay M. Webb, Hanna Cortado, Frank Robledo-Avila, Steve Oghumu, Abhay R. Satoskar, Miriam Rodriguez-Sosa, Luis I. Terrazas, Mireia Guerau-de-Arellano, Santiago Partida-Sánchez

**Affiliations:** 1 Department of Pathology, The Ohio State University, Columbus, OH 43221, USA; 2 Center for Microbial Pathogenesis, The Research Institute at Nationwide Children’s Hospital, Columbus, Ohio, USA; 3 School of Health and Rehabilitation Sciences, Medical Laboratory Science Division, The Ohio State University, Columbus, Ohio, USA; 4 Unidad de Biomedicina, Facultad de Estudios Superiores-Iztacala, UNAM, Tlalnepantla, MEX, Mexico; 5 Biomedical Sciences Graduate Program, The Ohio State University, Columbus, Ohio, USA; 6 Environmental Health Sciences, College of Public Health, Ohio State University, Columbus, OH, USA; 7 Department of Pediatrics, College of Medicine, The Ohio State University, Columbus, Ohio, USA

## Abstract

Helminths cause chronic infections and affect the immune response to unrelated inflammatory diseases. Although helminths have been used therapeutically to ameliorate inflammatory conditions, their anti-inflammatory properties are poorly understood. Alternatively activated macrophages (AAMϕs) have been suggested as the anti-inflammatory effector cells during helminth infections. Here, we define the origin of AAMϕs during infection with *Taenia crassiceps*, and their disease-modulating activity on the Experimental Autoimmune Encephalomyelitis (EAE). Our data show two distinct populations of AAMϕs, based on the expression of PD-L1 and PD-L2 molecules, resulting upon *T. crassiceps* infection. Adoptive transfer of Ly6C^+^ monocytes gave rise to PD-L1^+^/PD-L2^+^, but not PD-L1^+^/PD-L2^−^ cells in *T. crassiceps*-infected mice, demonstrating that the PD-L1^+^/PD-L2^+^ subpopulation of AAMϕs originates from blood monocytes. Furthermore, adoptive transfer of PD-L1^+^/PD-L2^+^ AAMϕs into EAE induced mice reduced disease incidence, delayed disease onset, and diminished the clinical disability, indicating the critical role of these cells in the regulation of autoimmune disorders.

Macrophages play a dual role during inflammation. While classical activation of macrophages promoted by interferon gamma (IFN-γ) or pathogen molecules [e.g., lipopolysaccharides (LPS)] contributes to pathogen elimination; tissue damage and inflammatory pathologies may ensue via inflammatory mediators such as nitric oxide and tumor necrosis factor alpha (TNF-α). Conversely, stimulation of macrophages with T helper cell type 2 (Th2) cytokines results in a phenotype [alternatively activated macrophages (AAMϕs)] involved in tissue repair and inflammation resolution^1^. The potential of harnessing this dichotomy in macrophage polarization to reprogram undesirable responses during inflammation requires a clear understanding of macrophage phenotype and function. Despite the well characterized polarization induced by IFN-γ or interleukin 4 (IL-4) *in vitro*^2^,^3^, macrophage polarization *in vivo* has proven more complex. The *in vivo* microenvironment contains a multitude of signals that influence the final reprograming of the macrophage population^1^,^4^. In addition, differential ontogeny among macrophage subsets adds further complexity to macrophage function.

Tissue resident macrophages originate before birth and acquire tissue specific phenotypes^5^,^6^,^7^. Tissue resident macrophages are maintained during adulthood mostly independent of blood-derived macrophages^8^,^9^. During inflammation however, blood-derived macrophages infiltrate the tissue, resulting in a mixture of resident and blood-derived macrophages^10^. The comparative responses of tissue and blood-derived macrophages and the functional role of each macrophage subset during inflammation remain to be investigated.

Helminth parasites induce a plethora of regulatory pathways to prevent elimination and allow long-term survival in their host. The immune response mounted against these parasites is accompanied by expansion and/or recruitment of regulatory B cells, Th2, T regulatory cells, eosinophils and AAMϕs^11^. The immunoregulation elicited by helminths also affects bystander infections and pathologies. Exposure to helminths and other microorganisms has been associated with reduced incidence of autoimmune and inflammatory diseases^12^,^13^,^14^. In rodent models, helminths exhibit a striking regulation of inflammatory and autoimmune diseases including experimental autoimmune encephalomyelitis (EAE), type 1 diabetes, colitis, asthma, allergies arthritis^15^, and colon cancer^16^. Likewise, helminths provided therapeutic improvement in patients suffering from multiple sclerosis (MS) and ulcerative colitis^17^,^18^,^19^. In addition, MS patients naturally infected with helminths had fewer relapses than uninfected patients, and elimination of the parasites worsened their condition^20^,^21^. Despite these findings, the use of helminths as a therapy has a number of associated risks^13^,^22^. Therefore, delineating the mechanisms involved in helminth-immunoregulation with the aim of designing immuno-therapies without the complications associated with the administration of the live parasite, is of increasing interest^23^.

EAE is an extensively used animal model of MS, an inflammatory demyelinating autoimmune disease characterized by progressive neurologic disability^24^. EAE is induced by immunization with adjuvants and myelin oligodendrocyte glycoprotein (MOG) peptides, which leads to the expansion of autoreactive T cells that secrete IFN-γ and IL-17 cytokines and express T-bet and RORγT transcription factors, respectively^25^,^26^,^27^,^28^. Autoreactive T cells infiltrate the central nervous system (CNS) and activate the microglia inducing the recruitment of inflammatory macrophages. Inflammatory macrophages are directly involved in the demyelination process^10^,^29^. In experimental models, different species of helminths have shown positive effects on EAE progression. This positive effect has been mainly attributed to the immune deviation from pathogenic Th1/Th17 responses towards a more beneficial Th2 immune response (Fleming, 2013).

A possible mechanism of immunoregulation by helminths is the induction of AAMϕs triggered by a Th2 microenvironment or helminth-derived molecules^16^,^30^. Helminth-induced AAMϕs have an important role in regulating the immune response and limiting inflammation. For example, during *Schistosoma manosoni* or *Nippostrongylus brasiliensis* infection, lack of AAMϕs results in enhanced tissue damage^31^,^32^. Additionally, AAMϕs generated during experimental cysticercosis or schistosomiasis greatly suppress the T cell response *in vitro*^33^,^34^. However, the origin of these suppressor macrophages during experimental cysticercosis is unknown.

In this study, we hypothesized that AAMϕs generated during experimental cysticercosis can reintroduce regulatory functions during autoimmunity. Therefore, we investigated the ontogeny of the suppressor macrophages generated during experimental cysticercosis, and tested their ability to down-regulate EAE. We found that blood Ly6C^+^ monocytes become AAMϕs due to persistent *T. crassiceps* infection. The expression of mannose receptor (MR; CD206), programmed death-ligand 2 (PD-L2), and the chemokine receptors CCR2/CX_3_CR1 distinguished helminth-induced AAMϕs from tissue resident macrophages. Furthermore, *T. crassiceps-*induced AAMϕs, exhibited strong regulatory function on T cells and reversed the clinical signs of EAE in a mouse model. Together, these findings point to helminth-induced AAMϕs as potential targets for the treatment of chronic inflammatory diseases.

## Results

### Dynamics of macrophage populations during *Taenia crassiceps* infection

Intraperitoneal infection with the metacestode *T. crassiceps* (larval stage) polarizes the immune response towards a Th2 profile accompanied by enhanced expression of genes associated with alternatively activated macrophages (AAMϕs)^35^,^36^,^37^. In addition to the canonical AAMϕs markers *Arg1, Chil3,* and *Retnla, T. crassiceps*-induced AAMϕs express the surface molecules mannose receptor (MR), the macrophage galactose-type lectin-1 (MGL1), and the suppressor molecules PD-L1 and PD-L2^33^,^38^. The origin of these macrophages during experimental cysticercosis remains unknown.

**Figure 1:**
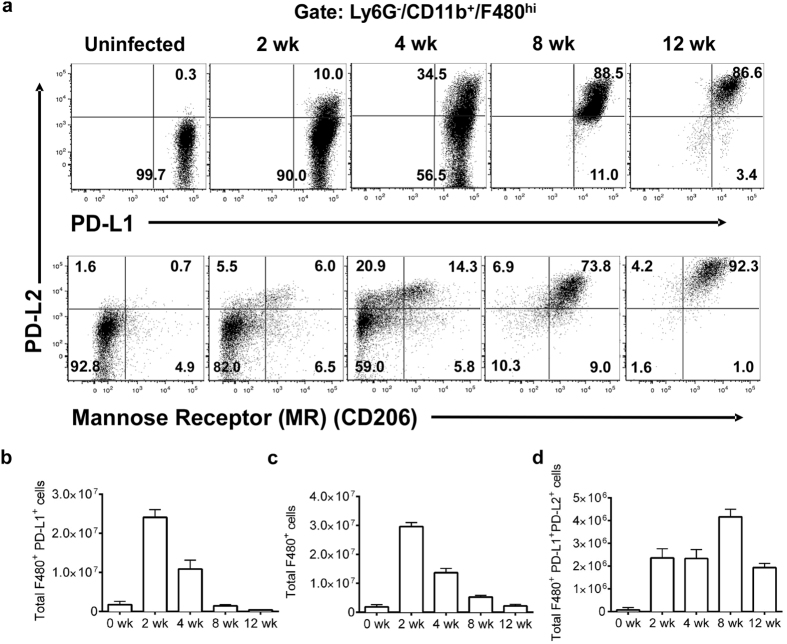
Macrophages acquire different phenotypes during *T. crassiceps* infection. C57BL/6 WT mice were *i.p.* infected with 40 metacestodes of *T. crassiceps* (ORF strain) and euthanized after 2, 4, 8 and 12 weeks. Peritoneal exudate cells were obtained from naïve or infected mice and analyzed for expression of PD-L1, PD-L2 and mannose receptor (MR) by flow cytometry. (**a**) Representative dot plots of the kinetic of AAMϕs in the peritoneal cavity. (**b**) Analysis of the absolute numbers of macrophages (CD11b^+^/F4/80^+^) after *T. crassiceps* infection. (**c**) Total number of PD-L1^+^/PD-L2^−^ macrophages. (**d**) Total number of PD-L1^+^/PD-L2^+^ macrophages during the course of the infection. Data are representative of 3 independent experiments (n = 3 animals at each time point) and are expressed as mean ± SE.

**Figure 2:**
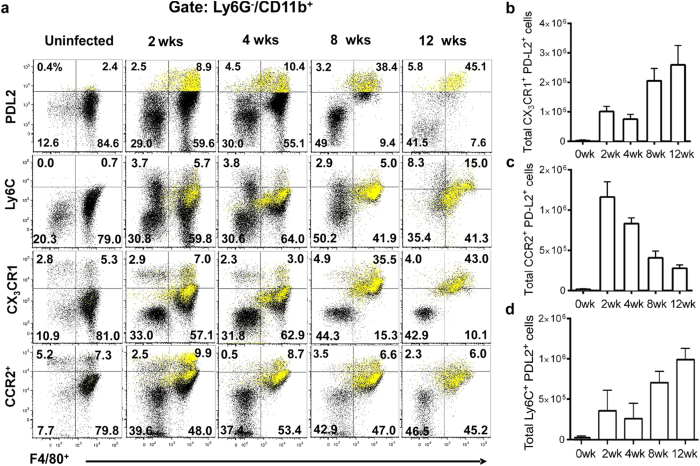
Heterogeneity of macrophage origin during *T. crassiceps* infection. C57BL/6 WT mice or C57BL/6 CX_3_CR1^gfp/+^ mice were infected with 40 metacestodes of *T. crassiceps* and euthanized after 2, 4, 8 and 12 weeks. PECs were recovered and analyzed by flow cytometry. CD11b^+^/PD-L2^+^ cells (yellow) were gated and overlaid in dot plots. (**a**) Representative dot plots of PD-L2, Ly6C, CCR2 and CX_3_CR1 expression in peritoneal macrophages during the course of infection. (**b**) Absolute numbers of CX_3_CR1^+^/PD-L2^+^, (**c**) CCR2^+^/PD-L2^+^ macrophages, and Ly6C^+^/PD-L2^+^ macrophages (**d**), after *T. crassiceps* infection. Data are representative of 3 independent experiments (n = 3 animals at each time point) and are expressed as mean ± SE.

By analyzing PD-L1, PD-L2, and MR (CD206) expression in macrophages (Supplementary Fig. 1), we found two distinct phenotypes of macrophages during experimental cysticercosis in the peritoneal cavity. In uninfected mice, resident macrophages constitutively expressed PD-L1, but not PD-L2 or MR (CD206). By 2-weeks post infection (wpi), macrophages expressing PD-L1, but not PD-L2 or MR, exceeded those in naïve mice. This macrophage population was subsequently reduced after 2-wpi as the infection progressed (Fig. 1a,c). At 2-wpi a small fraction of macrophages (10%; Figs 1a and 2 wpi) showed increased expression of PD-L2 (top) and MR (CD206; bottom). This population increased over time outnumbering the PD-L1^+^/PD-L2^−^ macrophages, representing more than 88% of peritoneal macrophages found at 8-wpi (Fig. 1a,d).

### Heterogeneity in macrophage origin during *T. crassiceps* infection

During Th1 type immune responses, blood-derived monocytes infiltrate the tissues and turn into classically activated macrophages (CAMϕs) or TNF-α and iNOS producing dendritic cells (TipDCs), which help to eliminate pathogens^39^. In contrast, under Th2 conditions (e.g., helminth infection or IL-4 administration), recruitment of blood-derived macrophages is scant. Instead, resident macrophages expand by proliferation^40^. This opened the question as to which population, tissue resident macrophages or blood monocytes, gives rise to the PD-L2^+^ macrophages during experimental cysticercosis. To answer this question, we used CX_3_CR1^+/gfp^ reporter mice, where monocytes and monocyte-derived cells, but not resident peritoneal resident macrophages, are labeled with green fluorescent protein (GFP)^41^. As previously reported^41^,^42^, we observed few macrophages expressing CX_3_CR1 in the peritoneal cavity of uninfected mice, consistent with most macrophages being CX_3_CR1^−^ resident macrophages (Fig. 2a). *T. crassiceps* infection caused a gradual increase in the number of F4/80^+^ cells expressing CX_3_CR1 until they constituted the majority of macrophages during the chronic infection (Fig. 2a). CX_3_CR1^+^ macrophages gained PD-L2 expression over the time. At 8 and 12 weeks, the majority of CX_3_CR1^+^ macrophages expressed PD-L2 (Fig. 2a,b).

The expression of CX_3_CR1 in macrophages strongly suggests infiltration of blood monocytes into the peritoneal cavity during *T. crassiceps* infection. Monocytes are subdivided into two different populations based on CX_3_CR1, CCR2 and Ly6C expression^43^. During bacterial and protozoan infections Ly6C^hi^/CCR2^+^/CX_3_CR1^low^/CD11b^+^ inflammatory monocytes infiltrate the tissue and give rise to CAMϕs or TipDCs, eventually down-regulating CCR2 and Ly6C expression^39^,^44^,^45^. In contrast, AAMϕs generated during the resolution of inflammation are currently thought to derive from Ly6C^−^/CCR2^−^/CX_3_CR1^hi^/CD11b^+^ patrolling monocytes^46^. To gain insight into the characteristics of infiltrating monocytes during *T. crassiceps* infection, we evaluated the expression of monocyte markers Ly6C and CCR2. Ly6C^+^ cells were scant in naïve mice (Fig. 2a). However, CD11b^+^/Ly6C^+^ cells were found in the peritoneal cavity at 2-wpi, and some expressed the macrophage marker F4/80 (Fig. 2a) At 8 and 12 weeks the majority of F480^+^PD-L2^+^ cells expressed medium levels of Ly6C, suggesting that a Ly6C^+^ population converts into mature macrophages during helminth infection (Fig. 2a). In addition, one population of macrophages remained expressing high levels of Ly6C, possibly indicating continuous recruitment of Ly6C^+^ cells into the pool of F480^+^PD-L2^+^ macrophages (Fig. 2d). With regards to CCR2 expression, CCR2^+^ cells represented 60% of the PD-L2^+^ macrophages at 2-wpi, however PD-L2^+^ macrophages gradually down-regulated CCR2 during chronic infection (Fig. 2a). The expression of CCR2 was inversely correlated with expression of CX_3_CR1 in PD-L2^+^ macrophages (Fig. 2a,c). Thus, during *T. crassiceps* infection, resident macrophages predominate in the early stages and do not express PD-L2, while monocyte-derived macrophages gain PD-L2 expression and represent the majority of macrophages during chronic infection.

### PD-L2^+^ macrophages are recruited in a CCR2 dependent manner

**Figure 3:**
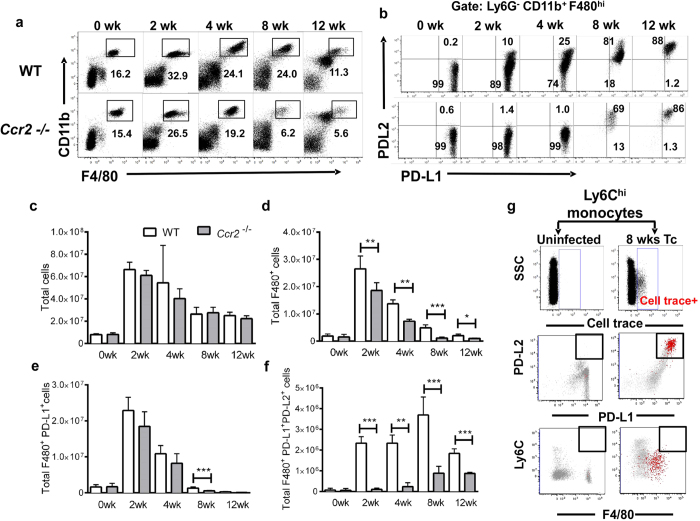
PD-L2^+^ macrophages require CCR2 expression to accumulate during *T. crassiceps* infection and are derived from Ly6C^hi^ monocytes. C57BL/6 WT mice and *Ccr2*^−/−^ mice were *i.p.* infected with 40 metacestodes of *T. crassiceps*, peritoneal cells were obtained after 2, 4, 8 and 12 weeks. The number of macrophages and the expression of PD-L1 and PD-L2 were evaluated by flow cytometry. (**a**) Representative dot plots of the kinetic of macrophage accumulation in WT *or Ccr2*^−/−^ infected mice. (**b**) Dot plots depicting the kinetic of PD-L1 and PD-L2 expression in macrophages (F4/80^+^CD11b^+^). (**c**) Analysis of total PECs in WT or *Ccr2*^−/−^ mice. (**d**) Absolut number of macrophages (F4/80^+^CD11b^+^), (**e**) Macrophages expressing PD-L1 and (**f**) macrophages expressing PD-L1 and PD-L2. Data are representative of 3 independent experiments (n = 3 animals at each time point) and are expressed as mean ± SE. (**g**) Intravenous adoptive transfer of cell trace labeled Ly6C^hi^ monocytes from bone marrow into naïve or 8 weeks *Taenia*-infected mice. Five days post-transfer, monocytes were identified as cell trace positive and overlaid in dot plots for the analysis of PD-L1, PD-L2, Ly6C and F4/80 expression. Data representative of 2 independent experiments (*n* = 2).

Based on our observations (Fig. 2a–c) we hypothesized that Ly6C^hi^ monocytes were recruited into the peritoneal cavity and differentiated into Ly6C^−^/CCR2^−^/CX_3_CR1^+^/PD-L2^+^ macrophages. Inflammatory Ly6C^+hi^ monocytes are known to egress from the bone marrow in a CCR2 dependent manner^47^. Once in the tissues they down-regulate Ly6C and CCR2 while up-regulating CX_3_CR1 expression^45^. Consequently, we expected that *Taenia*-induced monocyte recruitment would be impaired in *Ccr2*^−/−^ mice. *Ccr2*^−/−^ mice have normal numbers of resident macrophages, but display a profound defect in Ly6C^hi^ monocyte recruitment into the peritoneal cavity^48^. The total number of immune cells recruited into the peritoneal cavity during infection was similar in wild type (WT) and *Ccr2*^*−/−*^ mice (Fig. 3c).

While CCR2 deficiency had no effect on baseline numbers of peritoneal macrophages (Fig. 3a), the number of macrophages induced by the infection was significantly reduced in *Ccr2*^*−/−*^ vs. WT mice (Fig. 3a,d). Analysis of macrophage subpopulations revealed an unaltered number of resident F4/80^+^/PD-L1^+^/PD-L2^−^ macrophages in *Ccr2*^−/−^ mice (Fig. 3a,e). The number of monocyte-derived F4/80^+^/PD-L1^+^/PD-L2^+^ macrophages was severely affected in *Ccr2*^−/−^ mice at 2- and 4-weeks post infection. By 8- to 12-weeks post infection, we found PD-L2^+^ macrophages in *Ccr2*^−/−^ mice, albeit significantly fewer than in WT mice (Fig. 3f). These findings reveal a critical role for CCR2 in the accumulation of PD-L2^+^ macrophages during experimental cysticercosis, and strongly support the hypothesis that PD-L2^+^ macrophages originate from newly recruited Ly6C^hi^ inflammatory monocytes.

### Ly6C^hi^ monocytes are reprogrammed into PD-L2^+^ macrophages during *T. crassiceps* infection

To further confirm that Ly6C^hi^ monocytes give rise to PD-L2^+^ macrophages during *T. crassiceps* infection, fluorescently-labeled bone-marrow Ly6C^hi^ monocytes were intravenously transferred into naïve or *T. crassiceps* chronically infected mice. Five days post transfer, donor monocytes were found in the peritoneal cavity of infected, but not of uninfected, mice. Transferred monocytes down-regulated Ly6C expression becoming Ly6C^low^, while donor monocytes up-regulated PD-L2 and F4/80 expression at levels equivalent to those in host PD-L2^+^ macrophages (Fig. 3g). These data suggest that during *T. crassiceps* infection there is an active recruitment of Ly6C^hi^ monocytes into the peritoneal cavity that differentiate into PDL2^+^ macrophages.

Our data show a complex dynamic of macrophage populations during *T. crassiceps* infection. Although resident macrophages are predominant during acute infection, blood-derived macrophages surpass the number of resident macrophages during chronic infection. These two macrophage populations showed a differential response to the microenvironment reflected in the expression of MR and PD-L2 only in blood-derived macrophages (Figs 1 and 2).

### Blood-derived macrophages and tissue-resident macrophages become AAMϕs during experimental cysticercosis

**Figure 4:**
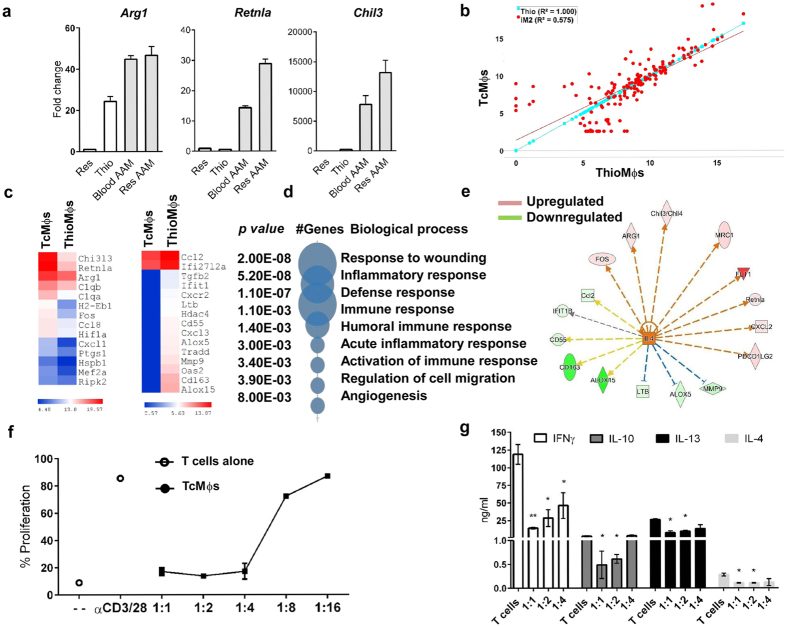
Blood-derived macrophages become alternatively activated during *T. crassiceps* infection and suppress Th1 and Th2 responses *in vitro*. Resident F4/80^+^/CX_3_CR1^−^ and blood-derived macrophages F4/80^+^/CX_3_CR1^+^ were sorted from 8 wks *Taenia*-infected mice (**a**) Evaluation of *Arginase1, Chil3* and *Retnla* expression by qPCR in naïve resident, thioglycollate elicited macrophages, and resident and blood-derived macrophages from *Taenia* infected mice. Data represents fold change over resident naïve macrophages. (**b**) Microarray comparison (Log2 transformed data) of 72 h thioglycollate (blue dots) elicited macrophages and blood-derived macrophages (red dots) from 8wks *Taenia*-infected mice. (**c**) Heat map of log 2 transformed counts of upregulated or downregulated genes (2 fold, *p* < *0.05*) identified in (**b**). (**d**) Biological processes associated with overexpressed genes in *Taenia crassiceps*-blood-derived macrophages using DAVID database. (**e**) Network of most downregulated and upregulated genes in *Taenia crassiceps* blood-derived macrophages vs thioglycollate macrophages and its association with IL-4 generated in Ingenuity pathway software. Red upregulated genes, green downregulated genes, Orange and blue arrows represent predicted activation or inhibition, respectively. (**f**) Naïve splenocytes were stimulated with anti CD3/CD28 and co-cultured with sorted blood-derived macrophages from *Taenia*-infected mice. (**f**) Proliferation of CD4+ cells evaluated by CFSE dilution after 72 h. (**g**) Cytokine production in macrophages/T cell co-cultures. In (**a–c**) macrophages were pooled from three mice and data represent two replicates. In (**f**,**g**) data are representative of one of two independent experiments using two replicates. Significance was calculated using t-test. *p < 0.05, **p < 0.01, ***p < 0.001.

PD-L2 and MR expression have been associated with alternative activation of the macrophages in response to IL-4^49^,^50^. Furthermore, a recent report showed that PD-L2 is differentially expressed on blood derived monocytes after IL-4 stimulation, but not in resident macrophages^51^. Since PD-L2 and MR molecules are expressed in blood-derived macrophages and are absent in resident macrophages during experimental cysticercosis, we questioned whether only blood-derived macrophages acquired an AAMϕs profile. To investigate the phenotype of macrophages upon *T. crassiceps* infection, we evaluated the expression of AAMϕs-associated genes in blood-derived and tissue resident macrophages isolated from the peritoneal cavity of mice 8 weeks after *T. crassiceps* infection. We found that both macrophage subsets overexpressed *Arg1, Chil3*, and *Retnla* compared with naïve resident peritoneal macrophages or thioglycollate elicited blood-derived macrophages (Fig. 4a). Thus, the microenvironment generated during experimental cysticercosis induces the expression of canonical AAMϕs genes in both resident and blood-derived macrophages.

Next, we performed nCounter gene expression analysis on blood-derived AAMϕs obtained from 8-weeks *Taenia*-infeced mice. Of the 250 immune-related genes assessed (Supplementary Table 1), 31 genes were dysregulated (2-fold change, p < 0.05) in PD-L2^+^ AAMϕs in comparison with thioglycollate elicited macrophages (Fig. 4c). As expected, PD-L2^+^ AAMϕs had higher *Arg1, Retnla* and *Chil3* expression than control macrophages. Additionally, PD-L2^+^ AAMϕs expressed other genes related with alternative activation such as *Flt1* and *Ptgs1*, involved in angiogenesis and prostaglandin production, respectively. PD-L2^+^ AAMϕs also showed increased expression of *Ccl8* and *Cxcl1*, chemokines involved in eosinophil and neutrophil recruitment, respectively. While PD-L2^+^ AAMϕs over-expressed the CAM-associated gene *Hifa,* it down-regulated molecules associated with lipid metabolism and alternative activation (*Alox5* and *Alox15*)^3^. Genes related with CAMs were down-regulated in PD-L2^+^ AAMϕs such as interferon induced genes (*Oas, Ifit*) and molecules involved in TNF (*Tradd*) signaling (Fig. 4c). To gain insight in the biological function of AAMϕs, we analyzed up-regulated genes using the DAVID Bioinformatics Resources 6.7 [National Institute of Allergy and Infectious Diseases (NIAID), NIH]^52^.

The biological processes associated with PD-L2^+^ AAMϕs aligned with the canonical functions reported for AAMϕs, such as wound healing and angiogenesis (Fig. 4d). Next, using Ingenuity Pathway Analysis (IPA) software, we generated a network of the significantly affected genes found in the gene expression array and molecules on the membrane of PD-L2^+^ AAMϕs identified by flow cytometry, and their relation with IL-4 (Fig. 4e). As expected, IL-4 was predicted to activate or down-regulate most of the genes we found regulated from our gene expression array of PD-L2^+^ AAMϕs. Finally, we evaluated the suppressor activity of PD-L2^+^ *macrophages* on pre-activated T cells *in vitro*. PD-L2^+^ macrophages suppressed proliferation of pre-activated T cells (Fig. 4f), in agreement with previous studies using unsorted AAMϕs isolated from *T. crassiceps* infected Balb/c mice^16^. Additionally, PD-L2^+^ macrophages down-regulated the production of both Th1 and Th2 associated cytokines in these cultures (Fig. 4g).

### Monocyte-derived *Taenia*-AAMϕs ameliorate the course of EAE

**Figure 5:**
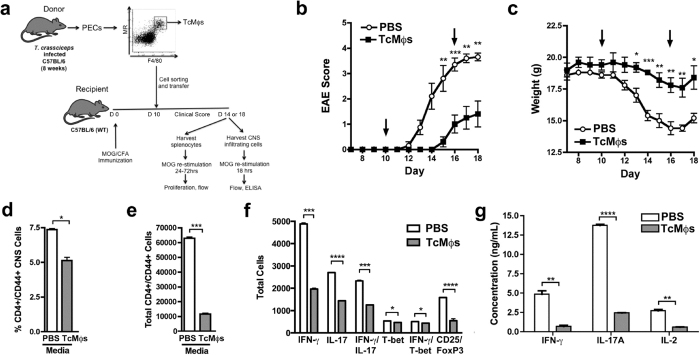
PD-L2^+^ macrophages from *T. crassiceps*-infected mice regulate the course of EAE. (**a**) Diagram of experimental design (mouse drawing adapted from ©Motifolio, Inc). (**b,c**) Mice were injected with CFA/MOG_35–55_ followed by pertussis toxin 2 and 24 hours later to induce EAE in 11-week female B6 mice. They were subsequently injected with PBS or macrophages isolated from mice infected with *Taenia crassiceps* on days 10 and 16 (arrows). The plots from one experiment are representative of two independent experiments. (**b**) EAE scores are shown as mean +/−SEM (see Table 1 for a more detailed analysis). (**c**) Mouse weights are measured in grams and shown as mean +/−SEM. (D-F) Cells were isolated from the brain and spinal cord of mice, treated with vehicle control or MOG_35–55_ peptide at 25 μg/mL, and processed for flow cytometry. (**d**) Percentage and (**e**) total number of CD4^+^/CD44^+^ cells found in the CNS. (**f**) Isolated cells from CNS were treated with MOG and gated on CD4^+^/CD44^+^ cells (IFN-γ, IL-17, IFN-γ/IL-17, T-bet, IFN-γ/T-bet) or CD25^+^/FoxP3^+^ cells (Tregs) and total number of IFN-γ^+^, IL-17^+^, IFN-γ^+^/IL-17^+^, T-bet^+^, IFN-γ^+^/T-bet^+^, or CD25^+^/FoxP3^+^ are shown. (**g**) Concentration of IFN-γ, IL-17A or IL-2 measured by ELISA. Significance was calculated using t-test. *p < 0.05, **p < 0.01, ***p < 0.001, ****p < 0.0001.

In EAE, pro-inflammatory myelin-specific auto-reactive Th1 and Th17 cells infiltrate the CNS, driving neuronal demyelination and causing ascending paralysis^24^. Pre-infection with *T. crassiceps* and other helminths down-regulates EAE disease^53^,^54^. To study the contribution of *T. crassiceps*-induced PD-L2^+^ AAMϕs to EAE disease suppression, we transferred purified macrophages to EAE mice. To avoid interfering with PD-L2, a regulatory molecule, PD-L2^+^ macrophages from chronically (8 week) *T. crassiceps-*infected mice were sorted based on F4/80 and MR expression since more than 95% of PD-L2^+^ macrophages co-express MR (Fig. 1a). We transferred sorted macrophages into mice 10 days after immunization with myelin oligodendrocyte glycoprotein (MOG) peptide (experimental design depicted in Fig. 5a), a stage at which myelin-specific Th1/Th7 responses have developed and EAE signs are developing^54^. Transfer of *T. crassiceps*-induced PD-L2^+^ AAMϕs into EAE-induced mice (TcMϕs → EAE) resulted in delayed EAE onset (Fig. 5b), and decreased incidence (Table 1). In addition, TcMϕs → EAE mice showed reduced ascending paralysis of the hind limbs and tail, as reflected by significant reduction in the total disease burden, as measured by the area under the curve (AUC), and day-18 mean clinical score (Fig. 5b and Table 1). Similarly, transfer of PD-L2^+^ AAMϕs significantly attenuated the weight loss associated with EAE (Fig. 5c).

Next, we investigated whether the transferred macrophages affected T cell infiltration in the CNS. TcMϕs → EAE mice showed less frequency and number of activated T helper cells (CD4^+^/CD44^+^) infiltrating the CNS (Fig. 5d,e). Administration of PD-L2^+^ AAMϕs significantly blunted the presence of IL-17 and IFN-γ producing CD4^+^/CD44^+^ cells, and CD25^+^/Foxp3^+^ regulatory T (Treg) cells (Fig. 5f), whereas T-bet^+^ and IFN-γ producing T-bet^+^ cells were also reduced in the CNS (Fig. 5f). We further analyzed the ability of CNS isolated cells to respond to MOG *ex vivo*. As expected, MOG greatly induced re-stimulation IFN-γ, IL-17 and IL-2 in the EAE-PBS control group. In contrast, CNS cells from TcMϕs → EAE mice produced low or undetectable levels of these cytokines (Fig. 5g).

**Figure 6:**
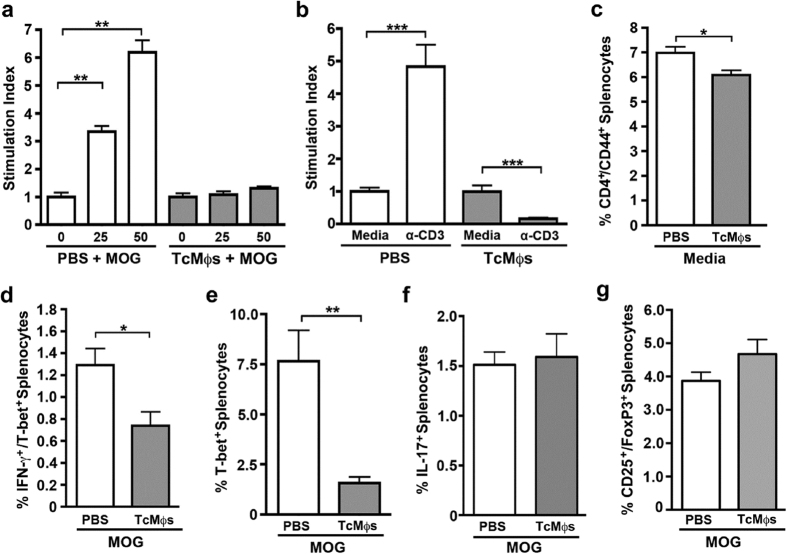
PD-L2^+^ *T. crassiceps* macrophages suppress the pathogenic T cell response in spleen. (**a**) Splenocytes from mice injected with either PBS or PD-L2^+^ macrophages (Tc-Mϕs) were treated with media containing vehicle control or with increasing concentrations of MOG_35–55_ peptide (25 and 50 μg/ml) and proliferation was measured by 3H-thymidine incorporation. (**b**) Splenocytes from EAE mice injected with either PBS as a control or *Taenia crassiceps*-sorted macrophages were plated with media alone or on anti-mouse CD3 coated plates and proliferation was measured by ^3^H-thymidine incorporation. Stimulation index is the cpm for MOG or anti-CD3 concentrations divided by cpm for media alone. (**c–f**) Splenocytes were analyzed by flow cytometry and CD4^+^/CD44^+^ cells were gated on to identify activated CD4^+^ helper T cells (**c**). The CD4^+^/CD44^+^ population was examined to identify the percentage of IFN-γ^+^/T-bet^+^ cells (**d**), T-bet^+^ cells (E), IL-17^+^ cells (**f**) and CD25^+^/FoxP3^+^ cells (**g**). Significance was calculated using ANOVA (**a**) or t-test (**b–f**). The *p* value was indicated as follows: *p < 0.05, **p < 0.01, ***p < 0.001.

When we investigated the T cell response in the spleen, a key organ for expansion of autoreactive T cells during EAE, splenocyte re-stimulation resulted in proliferation in response to MOG in EAE-PBS treated mice. In contrast, TcMϕs → EAE treated mice showed null proliferation in response to MOG concentrations at 25 and 50 μg/mL (Fig. 6a). To test whether nonspecific T cell response was also affected in TcMϕs → EAE mice, we stimulated splenocytes with anti-CD3, a polyclonal stimulus. Again, EAE induced mice proliferated in response to CD3 stimulation, however this response was absent in TcMϕs → EAE mice (Fig. 6b). Furthermore, transfer of PD-L2^+^ AAMϕs negatively affected the frequency of CD4^+^/CD44^+^ T cells and their ability to produce IFN-γ and express T-bet in the spleen (Fig. 6c–f). The frequency of IL-17^+^ cells and Foxp3^+^ cells was not affected by the presence of PD-L2^+^ macrophages in this organ (Fig. 6g,h). Further, IL-4 secretion was not detected in the CNS or the spleen after MOG re-simulation. Together these data show that PD-L2^+^ macrophages generated during *T. crassiceps* infection have a potent modulatory activity on the course of EAE by down-regulating the pathologic T cell response.

## Discussion

Our study shows that during experimental cysticercosis, Ly6C^hi^ monocytes are recruited into the peritoneal cavity in a CCR2-dependent manner giving rise to AAMϕs expressing PD-L2 and MR. Monocyte-derived AAMϕs differed from resident AAMϕs in PD-L2 and MR expression levels. Furthermore, adoptive transfer of PD-L2^+^ AAMϕs significantly suppressed autoreactive T cell response and EAE clinical disease. This is the first study showing a regulatory role for helminth-elicited AAMϕs on EAE.

The fact that Ly6C^hi^ monocytes become classically activated macrophages (CAMϕs) or TNF-α producing dendritic cells (TipDCs), and help to clear bacterial and protozoan infections, is widely accepted^39^,^46^,^55^. Here, we show that the microenvironment generated by the helminth *T. crassiceps* promoted the conversion of Ly6C^hi^ monocytes into AAMϕs with a suppressor phenotype. Recruitment of PD-L2^+^ AAMϕs is largely dependent on the chemokine receptor CCR2, corroborating their monocytic origin. Our data is in agreement with studies showing that Ly6C^hi^ monocytes become AAMϕs in the liver during schistosomiasis^56^,^57^. Similarly, Egawa *et al*. (2013) reported the conversion of Ly6C^+^ monocytes into regulatory AAMϕs in a skin allergy model^58^. These findings demonstrate Ly6C^+^ monocytes are an important source of AAMϕs during Th2 polarized conditions. An interesting study showed that during acute infection (<15 days) with the filarial parasite *Litomosoides sigmodontis* there was expansion of resident macrophages by proliferation induced by IL-4 in the pleural cavity, but limited recruitment of monocytes from the blood^40^. This phenomenon was parasite specific since thioglycollate administration recruited monocytes to the pleural cavity that were able to switch to AAMϕs after IL-4 administration^51^. In contrast, during chronic infection (>8wks) in the liver with the nematode *S. mansoni* blood derived macrophages were recruited and acquired an AAMϕ phenotype helping to form the granuloma^56^,^57^. Our data show that acute infection (<2wks) with the cestode *T. crassiceps* causes scant recruitment of blood derived macrophages. Interestingly during chronic infection (>8wks) blood derived macrophages are the main population of macrophages and acquire an AAMϕ phenotype. Thus differential sources of macrophages in various models of helminth infections (local expansion vs. recruitment) may account for the heterogeneity observed in AAMϕs function and phenotype during helminthic infections^59^. Then why do some helminthic infections trigger the recruitment of additional macrophages to the infection site? Resident tissue macrophages, which are unable to contain the infection, enhanced tissue damage, and/or persistent infection may account for this recruitment. Conversely, the initial Th1-like response during *T. crassiceps* infection^37^, may account for early recruitment of Ly6C^hi^ monocytes. Since *Ccr2*^*−/−*^ mice showed a profound defect in PD-L2^+^ macrophages at 2-wpi, this reinforces the idea that in *Ccr2*^*+/+*^mice an early recruitment of Ly6C^hi^ monocytes occurs during *T. crassiceps* infection. Subsequently, the early Th1 response shifts to a Th2 response around 4-wpi and is maintained over 12-wpi^37^. This Th2 polarized response is likely to be the cause of the reprogramming of Ly6C^hi^ recruited monocytes into AAMϕs during experimental cysticercosis since IL-4 enhances most of the up-regulated genes observed in this study.

Tissue-resident macrophages also adopted an AAMϕ phenotype during experimental cysticercosis in our study. However, they did not express PD-L2 or MR as did blood-derived macrophages. Gundra *et al*. also compared the gene expression of resting tissue peritoneal macrophages and 72 h thioglycollate elicited blood-derived macrophages stimulated with IL-4 *in vivo* finding different molecular signatures. Both macrophage subsets expressed AAMϕs related genes such as *Arg1*, yet with differential gene expression for MHCII, MR and PD-L2. In these settings both AAMϕs subsets proliferated in response to IL-4 and were able to suppress the T cell proliferation *in vitro*. Furthermore monocyte derived AAMϕs showed upregulated expression of genes related with immune response and resident AAMϕs upregulated genes related with metabolism^51^. However, some differences observed in the distinctive response to IL-4 may be the result of the specific microenvironment induced by thioglycollate, since resident macrophages were not exposed to such stimulus^51^. In our study however, blood- and tissue-derived AAMϕs were exposed to the same microenvironment generated by the infection. While both subsets expressed canonical AAMϕs markers, PD-L2 and MR expression differed. Our data reinforce the idea that blood-derived macrophages and tissue macrophages acquire distinct profiles under Th2 inflammation. The causes of such differential response and the resulting function of AAMϕs subsets require further investigation.

Our data show that blood-derived AAMϕs expressed genes related with angiogenesis and wound healing. Additionally, the expression of PD-L2 makes this population a candidate for immunoregulation. Indeed, PD-L2^+^ macrophages are associated with T cell anergy during *T. crassiceps* infection and blocked the T cell proliferation *in vitro*^33^. In addition, we demonstrated that both Th1 and Th2 cytokines were suppressed by the presence of *Taenia*-PD-L2^+^ macrophages. This aligns with previous observation where depletion of macrophages resulted in enhanced Th1 and Th2 cytokine production during experimental cysticercosis^60^. These data suggest that PD-L2^+^ AAMϕs, induced during cysticercosis, and probably other helminth infections, down-regulate the response of activated T helper cells disregarding the effector class. We speculate that these macrophages may also regulate Th2-related pathologies such as allergies and asthma.

Various studies have shown that pre-infected mice with helminth parasites, including *T. crassiceps*, develop attenuated EAE associated pathology^53^,^54^. In most of these studies EAE was induced in helminth pre-infected mice, or the mice were infected two or three days after EAE induction, with both cases resulting in attenuated pathology during EAE. However, helminth-infection during late stages of EAE had few or any beneficial effects, perhaps due to the time needed to induce suppressor macrophages and/or other regulatory cells after helminth infection^54^. The reduced pathology caused by helminths on the course of EAE has been largely attributed to immune deviation of autoreactive T cells towards a Th2 profile^54^. This could be the result of autoreactive T cell priming in a helminth-conditioned microenvironment characterized by a dominant Th2 response, which may favor the generation of non-pathogenic Th2 autoreactive cells.

These findings open two possibilities with respect to the therapeutic effect of these organisms. The first is that therapeutic infection with helminths could be less efficient in modulating inflammatory disease than pre-infection. Secondly, treatment with helminths may require a long time to efficiently induce regulatory mechanisms that can ameliorate inflammatory pathologies. For example, in *T. crassiceps* infection, scant numbers of PD-L2^+^ AAMϕs were detected at 2-wpi despite representing the dominant macrophage population after 8-wpi. In a co-infection model of *T. crassiceps* and the intracellular parasite *Trypanosoma cruzi*, the immune response to the latter was only modified in chronically infected *T. crassiceps* mice, but not in *T. crassiceps* acute infected mice^61^. Thus, modulation of the immune response by helminths to unrelated pathologies may require chronic infection with these organisms. This hypothesis is particularly difficult to test since most experimental models of inflammation are relatively short. In this context our data shows that PD-L2^+^ AAMϕs from chronically *T. crassiceps* infected mice had a striking effect on the course of EAE when they were injected after 10 days of EAE induction.

Thus regulatory cells from chronic helminth infection can be strong regulators of the immune response, suggesting that the therapeutic use of helminths may require increased time to exert regulatory functions on inflammatory-related pathologies. Administration of PD-L2^+^ macrophages did not result in immune deviation to a Th2 profile, but rather in a general suppression of T cell activation along with decreased T cell infiltration into the CNS and down-regulation of the pathologic cytokines IFN-γ and IL-17. The ability of PD-L2^+^ AAMϕs to down-regulate EAE may depend on the expression of PD-L1 and PD-L2, both related with EAE suppression^62^. We do not rule out additional regulatory factors. For example monocyte-derived AAMϕs showed overexpression of *Ptgs1*, a molecule involved in prostaglandin synthesis, which we have shown may account for the protective effect of *T. crassiceps* on colitis^16^. We did not find expression of the canonical regulatory cytokine IL-10 in PD-L2^+^ macrophages suggesting an IL-10 independent mechanism for T cell suppression.

Ly6C^hi^ monocytes in mice and their counterparts CD14^+^CD16^−^ population in humans have been involved in the pathogenesis of inflammatory diseases. Elegant studies have shown that avoiding recruitment of these subsets of monocytes can be an efficient strategy to modify ongoing inflammation^63^,^64^. Therefore, the transformation of Ly6C^hi^ cells into AAMϕs with the ability to regulate an exacerbated T cell response may represent a novel strategy to modify the phenotype of inflammatory monocytes that have already infiltrated the tissues during inflammatory diseases.

In conclusion, this study is the first to demonstrate that *T. crassiceps* infection recruits and converts Ly6C^+^ monocytes into immunoregulatory AAMϕs that suppress autoreactive T cell responses and EAE clinical disease. Therefore, PD-L2^+^AAMϕs are likely one of the mechanisms by which helminth infections ameliorate inflammatory and autoimmune disease. Finally, the novel finding that inflammation-associated Ly6C^hi^ monocytes can be re-programed into regulatory cells has important clinical implications for therapeutically targeting autoimmunity and exacerbated inflammation.

## Materials and Methods

### Ethics statement

All experiments in this study were performed according to the guidelines for the Care and Use of Laboratory Animals adopted by the US National Institutes of Health. The Institutional Animal Care and Use Committee (IACUC) at The Research Institute at Nationwide Children’s Hospital, and The Ohio State University approved all protocols. All efforts were made to minimize animal suffering during the course of these studies.

### Mice

Adult 6- to 8-wk female wild type (WT) mice (C57BL/6 background) and B6.129S4-*Ccr2*^*tm1Ifc*^/J (CCR2 KO) mice were purchased from The Jackson Laboratory. CX3CR1^*gfp/+*^ mice were obtained by crossing WT and B6.129P-*Cx3cr1*^*tm1Litt*^/J (CX3CR1^*gfp/gfp*^) mice both purchased from The Jackson Laboratory. Female 10-wk C57BL/6 mice were purchased from Taconic Biosciences, Inc. for EAE experiments. All animals were maintained in a pathogen-free environment and established as breeding colonies in the Transgenic Mouse Facility at The Research Institute at Nationwide Children’s Hospital animal facility or in specific pathogen-free conditions at The Ohio State University Laboratory Animal Resources. EAE experimental procedures were approved under Ohio State University’s IACUC protocol #2013A00000151 to ensure the humane care and use of animals.

### Parasites and experimental infection

Metacestodes of *T. crassiceps* (ORF strain) were harvested under sterile conditions from the peritoneal cavity of female C57BL/6 mice after 6–10 weeks of infection. The cysticerci were washed four times in physiological saline solution and used for infection. Because C57BL/6 mice are relatively resistant to the infection^53^, we infected WT, CCR2 KO and CX3CR1^gfp/+^ with 40 small non-budding cysticerci of *T. crassiceps* suspended in 0.3 mL of saline solution. The infected mice were euthanized at different time points and the peritoneal cavity cells were harvested and analyzed by flow cytometry.

### Flow cytometry analysis

Single-cell suspensions were obtained from peritoneal exudate cells (PECs) by peritoneal wash with 5-mL ice-cold phosphate buffer solution (PBS). PECs pellet were washed twice with complete Roswell Park Memorial Institute (RPMI) 1640 media and filtered with 70 μm nylon mesh (Fisher Scientific). ACK (Ammonium-Chloride-Potassium) Lysing Buffer (2-mL; GIBCO, Life technologies) was added to lyse red blood cells. Harvested cells were incubated in saturated doses of anti-mouse Fc receptor in 100-μl ice-cold FACS buffer (1% bovine serum albumin/0.01% sodium azide (NaN_3_) in PBS) for 15 min. After washing, 3 × 10^6^ cells were stained with various combinations of antibodies in ice-cold FACS buffer for 15 min, and further collected on a LSR II cytofluorometer (Becton Dickinson, BD). Cells were gated according to size (SSC-A) and forward scatter (FSC-A). Blue-fluorescent reactive dye L23105 (Life Technologies) was used to discard dead cells. Absolute cell numbers were calculated with the total cell count multiplied successively by the percentages for the appropriate gates obtained through analysis in FlowJo Software (FlowJo, LLC).

### Monocyte isolation and adoptive transfer

Monocytes were isolated as previously described^65^. Briefly, bone marrow cell suspensions were isolated by flushing femurs and tibias from 6- to 8-wk WT C57BL/6 mice with complete RPMI 1640 medium supplemented with 10% fetal calf serum (FCS), 100 units of penicillin/streptomycin, 2 mM glutamine, 25 mM HEPES buffer, and 1% non-essential amino acids (all from Gibco/Brl Division, Grand Island, NY, USA). Bone marrow mononuclear cells (BM-MNCs) were isolated using a density-gradient centrifugation method. BM-MNCs were incubated with a mixture of antibody microbeads according to the manufacturer’s protocol (Miltenyi Biotec, Auburn, CA). The cells were then run through a LD-negative selection column. The negative fraction was collected (putative monocytes) and stained for cell sorting with a BD Influx™ cell sorter. Isolated populations of Ly6C^high^ monocytes were stained for 20 min with the cell trace proliferation dye eFluor 450 and eFluor 670, respectively (eBioscience), Stained monocytes Ly6C^high^ were transferred (5 × 10^6^ cells each) intravenously into *T. crassiceps*-infected (8-wpi) or uninfected WT mice.

### RNA expression

Peritoneal cells from naïve, three days-thioglycollate induced PECs (intraperitoneal administration) or 8-wpi *Taenia*-infected mice were obtained by peritoneal lavage with 10 mL sterile PBS. Cells from two or three mice were pooled for each condition. Resident naïve macrophages (F4/80^hi^), thioglycollate elicited blood-derived macrophages (F4/80^+^/CX_3_CR1^+^), 8 weeks *Taenia* resident (F4/80^+^/CX_3_CR1-) or 8 weeks *Taenia* blood-derived macrophages (F4/80^+^/CX3CR1^+^) were sorted using FACs ARIA sorter at OSU. A dump channel was used to discard contaminating cells containing (anti-CD3, -CD19 and -Ly6G). Sorted cells were immediately transferred to TRizol and stored at −80 °C. RNA was extracted with regular TRizol protocol and QIAGEN columns were used to purify the RNA. RNA was quantified using the NanoDrop^TM^ spectrophotometer, and evaluation of RNA integrity was performed using the Agilent Bioanalyzer 2100. For quantitative polymerase chain reaction (qPCR), the cDNA synthesis kit (Bio-Rad) was used as per manufacturer instructions, and qPCR was performed in a Bio-Rad Thermo-Cycler using the Sybergreen kit (Bio-Rad) with primers from PrimerBank (Harvard). For NanoString analysis 50 ng/μL of RNA were analyzed using the Inflammatory set as per manufacturer instructions at the Genomic Shared Resource, OSU.

Data analyses were performed using nSolver™ Analysis Software. Background was subtracted using the geomean of negative controls +/−2 standard deviations (B–H) and normalized using the geomean of the housekeeping genes provided in the set. Negative control A was excluded from the analysis due to high background and per manufacturer suggestion. Probes with more than 40 counts were used to analyze significant changes in gene expression between samples. MultiExperiment Viewer (MeV) was used to generate heat maps, which represent Log_2_ transformed data^66^. Biological processes were obtained using the Database for Annotation, Visualization and Integrated Discovery (DAVID) v6.7^52^. Probes with >2-fold induction and *p* < *0.05* were uploaded to QIAGEN’S Ingenuity Pathway Analysis (IPA) to generate networks. Mannose receptor, PD-L2 and PD-L1 were manually added to the pathway.

### *In vitro* cell suppression assay

Peritoneal cells from *T. crassiceps* 8-wpi mice were obtained and labeled for F4/80 and MR. F4/80^+^MR^+^ were purified by FACScan™ flow cytometry exceeding 90% purity. Total splenoyctes were labeled with Carboxyfluorescein succinimidyl ester (CFSE) fluorescent cell staining dye and plated in anti-CD3/28 coated plates. Sorted F4/80^+^MR^+^ macrophages were plated in different ratios with total splenocytes. After 72 h, culture supernatants were recovered and stored at −80 °C for further cytokine quantification by enzyme-linked immunosorbent assay (ELISA). Cells were recovered and stained with anti-CD4, and cell proliferation was evaluated by flow cytometry. Data are presented as proliferation of CD4+ gated cells by CFSE dilution.

### EAE model

C57BL/6 female mice (11 weeks old) were immunized with a complete Freund’s adjuvant (CFA)/ MOG peptide emulsion along with pertussis toxin, (Hooke Laboratories, Lawrence, MS) according to manufacturer’s instructions. At 10- and 16-days post-immunization, mice intraperitoneally received 5 × 10^6^ sorted macrophages (F4/80^+^MR^+^) from *Taenia* infected mice (4–5 mice per group). EAE disease progression scoring was blinded and evaluated according to the Hooke Laboratories scoring guide: 0, no clinical disease; 1, limp tail; 2, moderate hind limb weakness; 3, complete hind limb paralysis; 4, quadriplegia or premoribund state; 5, death due to EAE.

At the peak of the disease mice were euthanized. Splenocytes and CNS cells were recovered by mashing the tissue through a 70 μM cell strainer. CNS cells were further processed using Percoll® gradients to enrich for immune cells as described^67^. Cells were plated with or without different concentrations of MOG (25 or 50 μg/mL) (Hooke Laboratories) or in coated plates with 1 μg/mL anti-CD3. After 48 h cells were pulsed with ^3^H-thymidine and at 72 h, cell proliferation was evaluated by measuring ^3^H-thymidine incorporation represented as Stimulation index (cpm for MOG or anti-CD3 concentrations divided by cpm for media alone). Cells were also examined by flow cytometry, and cytokine production was evaluated in supernatants by ELISA.

### Intracellular flow cytometry

Splenocytes and CNS cells were stimulated with MOG, and we evaluated the intracellular production of IFN-γ and IL-17, and T-bet expression. The presence of regulatory T cells (Treg), were determined by CD25 and FoxP3 expression. Cells were blocked with anti-mouse Fc receptor antibody (CD16/CD32, BD) for 15 min at 4 °C in FACS buffer (PBS with 2% FBS and 1 mM EDTA). Subsequently cells were surface stained with antibodies for anti-mouse CD4 (RM4-5, eBioscience) and CD44 (IM7, Biolegend) for cytokines and T-bet stainings or CD4 (RM4-5, eBioscience) and CD25 (PC61.5, eBioscience) for FoxP3 staining for 15 min at 4 °C. Cells were washed three times with FACS buffer, fixed with either eBioscience Foxp3 (staining reagents) or BD (IFN-γ, IL-17, T-bet staining) FIX & PERM® reagents (30 min, 4 °C) and blocked with Fc block for 15 min prior to staining with the intracellular antibodies (30–45 min, 4 °C) (anti-mouse IFN-γ (XMG1.2), anti-mouse IL-17 (TC11–18H10.1), anti-human T-bet (4B10) (all from BioLegend), FoxP3 (FJK-16s, eBioscience). Data were acquired on a BD FACSCanto^TM^ II Flow Cytometer (BD, NJ). Data were analyzed with FlowJo single cell analysis software.

### Statistics

Student t-test or ANOVA statistical analysis was performed for the experiments using Prism GraphPad Software; *p* values below 0.05 were considered significant.

## Additional Information

**How to cite this article**: Terrazas, C. *et al*. Helminth-induced Ly6C^hi^ monocyte-derived alternatively activated macrophages suppress experimental autoimmune encephalomyelitis. *Sci. Rep.* **7**, 40814; doi: 10.1038/srep40814 (2017).

**Publisher's note:** Springer Nature remains neutral with regard to jurisdictional claims in published maps and institutional affiliations.

## Supplementary Material

Supplementary material

## Figures and Tables

**Table 1:** Summary of clinical signs and EAE disease burden after adoptive transfer of AAMϕs from *T. crassiceps*-infected mice.

	PBS (control)	Tc-Μϕs	Δ/control	*p-value
% EAE	100.0	80.0	−20.0%	n/a
Average Day of Onset ± SD	13.00 ± 0.71	15.80 ± 0.84	2.8 Days	0.0107
Mean Score D18	3.65 ± 0.34	1.40 ± 1.14	−2.25	0.0114
Area Under the Curve	14.7 ± 3.25	3.25 ± 2.57	−11.45	0.0079
Max Score	4	3	−1	n/a
Mean Weight D10–18	17.0 ± 1.8	18.9 ± 0.7	+1.9 g	0.0013
Mean Weight D18	15.2 ± 0.8	18.3 ± 2.1	+3.1 g	0.0126
n	5	5	n/a	n/a

^*^Statistics tests. Day of Onset, Mean Score and AUC: Mann-Whitney or Unpaired *t*-test. Mean Weights: Unpaired *t*-test.
