# Supplementary material for: Helminth-induced Ly6Chi monocyte-derived alternatively activated macrophages suppress experimental autoimmune encephalomyelitis
Source: Sci Rep. 2017 Jan 17;7:40814. doi: 10.1038/srep40814 (PMC5240103; doi:10.1038/srep40814)
Supplement: Supplementary material [file srep40814-s1.pdf]

Helminth-induced Ly6C^{hi} monocyte-derived alternatively activated macrophages suppress experimental autoimmune encephalomyelitis

Cesar Terrazas, Juan de Dios Ruiz-Rosado, Stephanie A. Amici, Kyle A. Jablonski, Diana Martinez-Saucedo, Lindsay M. Webb, Hanna Cortado, Frank Robledo-Avila, Steve Oghumu, Abhay Satoskar, Miriam Rodriguez-Sosa, Luis I. Terrazas, Mireia Guerau-de-Arellano, and Santiago Partida-Sánchez

SUPLEMENTAL MATERIAL

Supplementary Figure 1. Gating strategy for macrophage identification. Peritoneal exudate cells (PECs) were isolated from uninfected or infected mice and analyzed by flow cytometry. Macrophage populations were analyzed by gating out debris, doublets, dead cells and neutrophils and gating in F480$^+$ CD11b$^+$ region. Subsequent analysis of different markers was analyzed as indicated in the corresponding figure's legend.

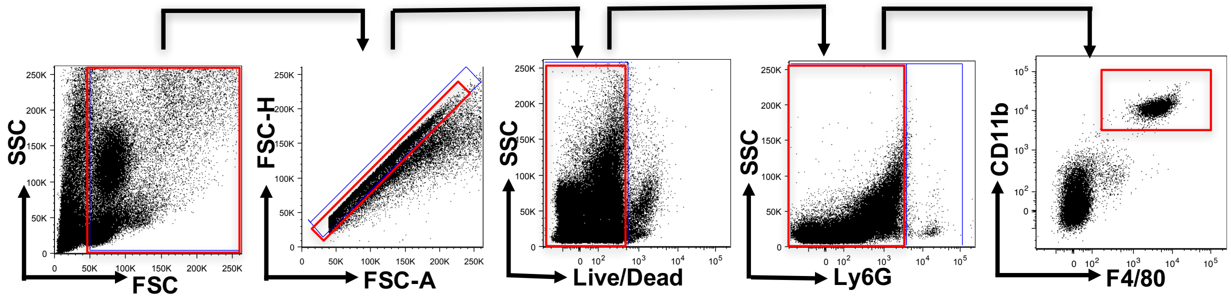

Supplementary Table 1. Nanostring analysis of *Taenia* elicited and Thioglycollate elicited macrophages presenting counts or log 2 data of genes expressed above the background levels. List of total gene expression evaluated in the array.

Gene Name	Accession #	**TcMϕs** counts	**ThioMϕs** counts	**Fold induction** **TcMϕs** vs ThioMϕs	**P value TcMϕs** vs ThioMϕs
Ager	NM_007425.2	5.95	2.43	2.45	0.496
Alox12	NM_007440.4	5.95	1	5.95	0.0384
Alox15	NM_009660.3	5.95	313.68	-52.68	0.0091
Alox5	NM_009662.2	5.95	70.83	-11.89	0.0036
Areg	NM_009704.3	5.95	1	5.95	0.0384
Arg1	NM_007482.3	315095.66	129771.76	2.43	0.0173
Atf2	NM_001025093.1	878.66	798.12	1.1	0.1678
Bcl2l1	NM_009743.4	108.75	519.65	-4.78	0.1078
Bcl6	NM_009744.3	13.09	63.88	-4.88	0.2444
Birc2	NM_007465.2	503.53	355.64	1.42	0.1554
C1qa	NM_007572.2	29982.45	7444.84	4.03	0.0198
C1qb	NM_009777.2	73634.2	27714.68	2.66	0.0029
C1ra	NM_023143.3	19.81	32.03	-1.62	0.7355
C1s	NM_144938.2	5.95	3.39	1.76	0.4251
C2	NM_013484.2	5.95	1	5.95	0.0384
C3	NM_009778.2	3214.74	428.5	7.5	0.056
C3ar1	NM_009779.2	18029.42	18136.27	-1.01	0.9777
C4a	NM_011413.2	1910.28	1309.55	1.46	0.5602
C6	NM_016704.2	23.69	1	23.69	0.0623
C7	XM_356827.6	63.87	1	63.87	0.0762
C8a	NM_146148.1	5.95	1	5.95	0.0384
C8b	NM_133882.2	5.95	1	5.95	0.0384
C9	NM_013485.1	5.95	1	5.95	0.0384
Ccl11	NM_011330.3	5.95	1	5.95	0.0384
Ccl17	NM_011332.2	5.95	10.7	-1.8	0.196

Ccl19	NM_011888.2	5.95	1	5.95	0.0384
Ccl2	NM_011333.3	3835.99	14987.64	-3.91	0.0122
Ccl20	NM_016960.1	5.95	4.68	1.27	0.2674
Ccl21a	NM_011124.4	10.1	1	10.1	0.1708
Ccl22	NM_009137.2	73.31	119.09	-1.62	0.3881
Ccl24	NM_019577.4	6610.39	5947.03	1.11	0.6062
Ccl3	NM_011337.1	454.33	1329.71	-2.93	0.1443
Ccl4	NM_013652.1	16.71	106.17	-6.35	0.3515
Ccl5	NM_013653.1	134.85	122.47	1.1	0.9157
Ccl7	NM_013654.2	1742.29	2312.42	-1.33	0.103
Ccl8	NM_021443.2	13136.52	4606.89	2.85	0.0464
Ccr1	NM_009912.4	1619.19	2035.91	-1.26	0.0821
Ccr2	NM_009915.2	6565.78	3462.12	1.9	0.0245
Ccr3	NM_009914.4	5.95	37.15	-6.24	0.0371
Ccr4	NM_009916.2	5.57	1	5.57	0.015
Ccr7	NM_007719.2	5.95	7.51	-1.26	0.2418
Cd163	NM_053094.2	5.95	272.29	-45.73	0.0175
Cd4	NM_013488.2	5.95	1	5.95	0.0384
Cd40	NM_011611.2	706.56	570.77	1.24	0.4601
Cd40lg	NM_011616.2	5.95	1	5.95	0.0384
Cd55	NM_010016.2	5.95	65.32	-10.97	0.0056
Cd86	NM_019388.3	530.83	584.38	-1.1	0.7671
Cdc42	NM_009861.1	37180.61	42959.33	-1.16	0.1305
Cebpb	NM_009883.3	11256.49	5988.9	1.88	0.0929
Cfb	NM_008198.2	1847.79	811.41	2.28	0.3562
Cfd	NM_013459.1	5.95	1	5.95	0.0384
Cfl1	NM_007687.5	15809.32	26083.56	-1.65	0.0104
Chi3l3	NM_009892.1	659942.94	15440.7	42.74	0.0147
Creb1	NM_133828.2	5.95	2.01	2.97	0.3582
Crp	NM_007768.4	5.95	1	5.95	0.0384
Csf1	NM_001113530.1	11.72	122.93	-10.49	0.1942
Csf2	NM_009969.4	5.95	1	5.95	0.0384
Csf3	NM_009971.1	5.95	1	5.95	0.0384
Cxcl1	NM_008176.1	1223.92	44.3	27.62	0.041

Cxcl10	NM_021274.1	5.95	1	5.95	0.0384
Cxcl2	NM_009140.2	4900.14	292.09	16.78	0.084
Cxcl3	NM_203320.2	5.95	66.61	-11.19	0.0048
Cxcl5	NM_009141.2	5.95	1	5.95	0.0384
Cxcl9	NM_008599.2	124.81	1	124.81	0.1075
Cxcr1	NM_178241.4	5.95	1	5.95	0.0384
Cxcr2	NM_009909.3	5.95	47	-7.89	0.0053
Cxcr4	NM_009911.3	925.87	591.21	1.57	0.0252
Cysltr1	NM_021476.4	1741.15	1425.98	1.22	0.2264
Cysltr2	NM_001162412.1	5.95	21.07	-3.54	0.0537
Daxx	NM_007829.3	17.44	116.43	-6.68	0.2977
Ddit3	NM_007837.3	250.12	558.83	-2.23	0.0358
Defa-rs1	NM_007844.2	5.95	2.01	2.97	0.3582
Elk1	NM_007922.4	5.95	16.06	-2.7	0.1373
Fasl	NM_010177.3	5.95	1	5.95	0.0384
Flt1	NM_010228.3	498.72	1	498.72	0.0022
Fos	NM_010234.2	12091.16	1079.91	11.2	0.0438
Fxyd2	NM_052823.2	6.65	47.48	-7.14	0.0541
Gnaq	NM_008139.5	1033.88	634.44	1.63	0.0709
Gnas	NM_010309.3	2203.78	1742.78	1.26	0.1256
Gnb1	NM_008142.3	10885.26	12254.83	-1.13	0.0381
Gngt1	NM_010314.2	5.95	1	5.95	0.0384
Gpr44	NM_009962.2	5.95	1	5.95	0.0384
Grb2	NM_008163.3	2207.2	1914.71	1.15	0.0476
H2-Ea-ps	NM_010381.2	5.95	1	5.95	0.0384
H2-Eb1	NM_010382.2	9507.8	382.68	24.85	0.0046
Hc	NM_010406.1	5.95	1	5.95	0.0384
Hdac4	NM_207225.1	5.95	52.6	-8.83	0.0312
Hif1a	NM_010431.2	13042.11	5617.49	2.32	0.0114
Hmgb1	NM_010439.3	1187.32	1005.76	1.18	0.2521
Hmgb2	NM_008252.3	5.95	32.3	-5.42	0.0192
Hmgn1	NM_008251.3	809.4	898.81	-1.11	0.3639
Hras1	NM_008284.2	5.95	1	5.95	0.0384
Hsh2d	NM_197944.1	5.95	2.72	2.19	0.143

Hspb1	NM_013560.2	166.15	22.38	7.42	0.046
Hspb2	NM_024441.3	5.95	1	5.95	0.0384
Ifi27l2a	NM_029803.1	2685.56	10624.58	-3.96	0.0258
Ifi44	NM_133871.2	13.15	1	13.15	0.2139
Ifit1	NM_008331.2	5.95	41.42	-6.96	0.0066
Ifit2	NM_008332.2	124.2	44.72	2.78	0.1405
Ifit3	NM_010501.1	202.4	137.38	1.47	0.0643
Ifna1	NM_010502.2	5.95	2.68	2.22	0.5653
Ifnb1	NM_010510.1	5.95	1	5.95	0.0384
Ifng	NM_008337.1	381.71	2.43	156.96	0.1107
Iigp1	NM_021792.3	377.51	12.23	30.86	0.1533
Il10	NM_010548.1	5.95	1.68	3.55	0.2347
Il10rb	NM_008349.5	5638.38	8253.29	-1.46	0.1337
Il11	NM_008350.2	82.03	61.6	1.33	0.5226
Il12a	NM_008351.1	5.95	1	5.95	0.0384
Il12b	NM_008352.1	5.95	1	5.95	0.0384
Il13	NM_008355.2	5.95	1	5.95	0.0384
Il15	NM_008357.1	14.43	115.39	-8	0.2827
Il17a	NM_010552.3	5.95	1	5.95	0.0384
Il18	NM_008360.1	152.05	168.12	-1.11	0.8435
Il18rap	NM_010553.2	5.95	8.48	-1.42	0.5824
Il1a	NM_010554.4	739.68	342.84	2.16	0.2005
Il1b	NM_008361.3	330.84	72.95	4.54	0.0552
Il1r1	NM_001123382.1	5.95	2.56	2.33	0.5325
Il1rap	NM_008364.2	248.55	274.94	-1.11	0.1682
Il1rn	NM_031167.4	164.21	442.85	-2.7	0.0502
Il2	NM_008366.3	5.95	1.23	4.83	0.043
Il21	NM_021782.2	5.95	1	5.95	0.0384
Il22	NM_016971.1	5.57	1	5.57	0.015
Il22ra2	NM_178258.5	5.95	1	5.95	0.0384
Il23a	NM_031252.1	5.95	1	5.95	0.0384
Il23r	NM_144548.1	5.95	1	5.95	0.0384
Il3	NM_010556.4	5.95	1	5.95	0.0384
Il4	NM_021283.1	5.95	1	5.95	0.0384

Il5	NM_010558.1	5.95	1	5.95	0.0384
Il6	NM_031168.1	20.52	1	20.52	0.2666
Il6ra	NM_010559.2	369.61	336.3	1.1	0.5516
Il7	NM_008371.2	8.15	10.67	-1.31	0.6385
Il9	NM_008373.1	8.15	1	8.15	0.1263
Irf1	NM_008390.1	2978.38	613.37	4.86	0.0855
Irf3	NM_016849.3	5.95	1	5.95	0.0384
Irf5	NM_012057.3	1556.3	1738.57	-1.12	0.1707
Irf7	NM_016850.2	223.24	667.25	-2.99	0.0973
Itgb2	NM_008404.4	7293.92	7452.64	-1.02	0.7778
Jun	NM_010591.2	3679.79	1992.36	1.85	0.1687
Keap1	NM_016679.4	351.7	267.52	1.31	0.0156
Kng1	NM_023125.3	5.95	3.21	1.85	0.6899
Limk1	NM_010717.2	229.06	119.06	1.92	0.0062
Lta	NM_010735.1	5.57	4.68	1.19	0.7912
Ltb	NM_008518.2	5.95	52.17	-8.76	0.0081
Ltb4r1	NM_008519.2	114.39	226.08	-1.98	0.0417
Ltb4r2	NM_020490.2	5.95	1	5.95	0.0384
Ly96	NM_016923.1	1314.99	1831.41	-1.39	0.0202
Maff	NM_010755.3	488.23	18.26	26.73	0.05
Mafg	XM_001002362.1	54.48	87.24	-1.6	0.3923
Mafk	NM_010757.2	35.39	49.44	-1.4	0.7203
Map2k1	NM_008927.3	2135.87	2173.27	-1.02	0.7811
Map2k4	NM_009157.4	785.7	886.91	-1.13	0.0771
Map2k6	NM_011943.2	5.95	4.28	1.39	0.584
Map3k1	NM_011945.2	437.59	642.06	-1.47	0.2412
Map3k5	NM_008580.4	157.97	274.83	-1.74	0.1753
Map3k7	NM_172688.2	1015.1	1381.06	-1.36	0.1097
Map3k9	NM_177395.4	5.95	1	5.95	0.0384
Mapk1	NM_001038663.1	713.3	816.62	-1.14	0.2763
Mapk14	NM_011951.2	690.12	814.47	-1.18	0.1873
Mapk3	NM_011952.2	1735.32	1326.21	1.31	0.3627
Mapk8	NM_016700.3	340.44	322.53	1.06	0.3254
Mapkapk2	NM_008551.1	738.32	1024.18	-1.39	0.1245

Mapkapk5	XM_990515.1	5.95	1.67	3.57	0.2307
Masp1	NM_008555.2	5.95	1	5.95	0.0384
Masp2	NM_010767.3	5.95	1	5.95	0.0384
Max	NM_008558.1	153.17	217.99	-1.42	0.0452
Mbl2	NM_010776.1	5.95	1	5.95	0.0384
Mef2a	XM_976032.1	369.07	49.59	7.44	0.0036
Mef2b	NM_001045484.1	5.95	1	5.95	0.0384
Mef2c_Mm	NM_025282.2	132.29	317.38	-2.4	0.1188
Mef2d	NM_133665.3	142.87	158.01	-1.11	0.6878
Mknk1	NM_021461.4	298.09	516.66	-1.73	0.0637
Mmp3	NM_010809.1	5.95	1	5.95	0.0384
Mmp9	NM_013599.2	5.95	94.65	-15.9	0.0029
Mrc1	NM_008625.1	14315.58	10917.57	1.31	0.1886
Mx1	NM_010846.1	443.93	166.69	2.66	0.1735
Mx2	NM_013606.1	5.95	4.75	1.25	0.6066
Myc	NM_010849.4	382.95	458.76	-1.2	0.0277
Myd88	NM_010851.2	890.53	805.17	1.11	0.4825
Myl2	NM_010861.3	5.95	9.71	-1.63	0.0851
Nfatc3	NM_010901.2	205.18	277.53	-1.35	0.0178
Nfe2l2	NM_010902.3	5092.36	6124.82	-1.2	0.0468
Nfkb1	NM_008689.2	1086.87	531.42	2.05	0.077
Nlrp3	NM_145827.3	81.54	47.84	1.7	0.604
Nod1	NM_172729.2	13.15	45.32	-3.45	0.3922
Nod2	NM_145857.2	5.95	23.69	-3.98	0.0559
Nos2	NM_010927.3	72.72	2.01	36.23	0.0765
Nox1	NM_172203.1	5.95	1	5.95	0.0384
Nr3c1	NM_008173.3	1087.88	1033.13	1.05	0.7245
Oas1a	NM_145211.2	1303.72	2294.47	-1.76	0.0009
Oas2	NM_145227.2	5.95	137.39	-23.07	0.0205
Oasl1	NM_145209.2	452.95	565.25	-1.25	0.1296
Pdgfa	NM_008808.3	411.17	162.59	2.53	0.213
Pik3c2g	NM_011084.2	96.14	2.43	39.53	0.1456
Pla2g4a	NM_008869.2	405.22	310.62	1.3	0.1565
Plcb1	NM_019677.1	444.07	662.03	-1.49	0.4907

Ppp1r12b	NM_001081307.1	10.1	10.29	-1.02	0.9818
Prkca	NM_011101.3	5.95	9.41	-1.58	0.1166
Prkcb	NM_008855.2	817.46	554.67	1.47	0.0741
Ptger1	NM_013641.2	5.95	1	5.95	0.0384
Ptger2	NM_008964.4	608.85	623.35	-1.02	0.9097
Ptger3	NM_011196.2	5.95	1	5.95	0.0384
Ptger4	NM_008965.1	255.26	149.21	1.71	0.0926
Ptgfr	NM_008966.3	5.95	1	5.95	0.0384
Ptgir	NM_008967.3	187.93	107.26	1.75	0.0812
Ptgs1	NM_008969.3	1155.52	211.76	5.46	0.0162
Ptgs2	NM_011198.3	41.63	1	41.63	0.3203
Ptk2	NM_007982.2	15.61	215.88	-13.83	0.2466
Rac1	NM_009007.2	9085.05	8883.57	1.02	0.3345
Raf1	NM_029780.3	479.71	704.94	-1.47	0.1716
Rapgef2	NM_001099624.2	321.65	384.8	-1.2	0.1773
Rela	NM_009045.4	628.48	519.3	1.21	0.327
Relb	NM_009046.2	528.6	480.99	1.1	0.1611
Retnla	NM_020509.3	777951.38	26534.99	29.32	0.0074
Rhoa	NM_016802.4	13158.27	8884.48	1.48	0.0077
Ripk1	NM_009068.3	315.99	456.39	-1.44	0.2138
Ripk2	NM_138952.3	332.81	161.35	2.06	0.0412
Rock2	NM_009072.2	73.64	168.23	-2.28	0.334
Rps6ka5	NM_153587.2	10.1	34.74	-3.44	0.2959
Shc1	NM_011368.4	537.79	403.37	1.33	0.0483
Smad7	NM_001042660.1	5.95	13.1	-2.2	0.0427
Stat1	NM_009283.3	1152.55	544.14	2.12	0.2656
Stat2	NM_019963.1	72.47	218.8	-3.02	0.5154
Stat3	NM_213659.2	2399.63	1244.44	1.93	0.0285
Tbxa2r	NM_001277265.1	5.95	1	5.95	0.0384
Tcf4	NM_013685.1	624.16	523.5	1.19	0.155
Tgfb1	NM_011577.1	3829.94	3357.16	1.14	0.3551
Tgfb2	NM_009367.1	5.95	40.83	-6.86	0.0074
Tgfb3	NM_009368.2	17.1	1	17.1	0.205
Tgfbr1	NM_009370.2	711.14	491.07	1.45	0.1364

Tlr1	NM_030682.1	748.53	1399.73	-1.87	0.0272
Tlr2	NM_011905.2	517.03	143.07	3.61	0.1162
Tlr3	NM_126166.2	5.95	9.92	-1.67	0.1266
Tlr4	NM_021297.2	2984.2	2003.73	1.49	0.0527
Tlr5	NM_016928.2	5.95	1	5.95	0.0384
Tlr6	NM_011604.3	609.39	626.29	-1.03	0.2302
Tlr7	NM_133211.3	211.71	467.38	-2.21	0.0672
Tlr8	NM_133212.2	3193.04	2853.66	1.12	0.148
Tlr9	NM_031178.2	10.1	17.11	-1.69	0.5562
Tnf	NM_013693.1	12.27	28.01	-2.28	0.408
Tnfaip3	NM_009397.2	4645.48	958.85	4.84	0.0944
Tnfsf14	NM_019418.2	62.86	83.15	-1.32	0.251
Tollip	NM_023764.3	1126.84	1252.23	-1.11	0.4927
Tradd	NM_001033161.2	5.95	74.9	-12.58	0.0094
Traf2	NM_009422.2	134.33	120.06	1.12	0.5248
Trem2	NM_031254.2	3065.36	2926.91	1.05	0.6731
Tslp	NM_021367.1	5.95	4.74	1.26	0.6157
Twist2	NM_007855.2	10.73	2.16	4.97	0.2426
Tyrobp	NM_011662.2	29167.97	33890.2	-1.16	0.0221
